# The experiences of close persons caring for people with chronic kidney disease stage 5 on conservative kidney management: Contested discourses of ageing

**DOI:** 10.1177/1363459314524805

**Published:** 2014-11

**Authors:** Joe Low, Jason Myers, Glenn Smith, Paul Higgs, Aine Burns, Katherine Hopkins, Louise Jones

**Affiliations:** University College London, UK; Imperial College London, UK; University College London, UK; Royal Free Hampstead NHS Trust, UK; University College London, UK

**Keywords:** chronic kidney disease, conservative kidney management, informal care, normal ageing, qualitative

## Abstract

Chronic kidney disease stage 5 is a global health challenge in the context of population ageing across the world. The range of treatment options available to patients at all ages has increased and includes transplantation and dialysis. However, these options are often seen as inappropriate for older frailer patients who are now offered the option of conservative kidney management, which is presented as a non-invasive alternative to dialysis, involving symptom management and addressing psychosocial needs. In this study, we conducted qualitative interviews with 26 close persons caring for someone with chronic kidney disease stage 5 in the United Kingdom to investigate how conservative kidney management interacted with implicit ideas of ageing, in both the experience of conservative kidney management and the understanding of the prognosis and future care of the kidney disease. Our findings highlighted participant confusion about the nature of conservative kidney management, which stems from an initial lack of clarity about how conservative kidney management differed from conventional treatments for chronic kidney disease stage 5. In particular, some respondents were not aware of the implicit palliative nature of the intervention or indeed the inevitable end-of-life issues. Although these findings can be situated within the context of communication failure, we would further argue that they also bring to the surface tensions in the discourses surrounding ageing and old age, drawing on the use of a ‘natural’ and a ‘normal’ paradigm of ageing. In the context of chronic kidney disease stage 5, more patients are being dialysed at older ages, but conservative kidney management is being advanced as a better option than dialysis in terms of quality of life and experience. However, in doing so, conservative kidney management implicitly draws on a notion of older age that echoes natural ageing rather than advocate a more interventionist approach. The role of discourses of ageing in the provision of treatments for conservative kidney management has not previously been acknowledged, and this article addresses this gap.

## Introduction

The nature of ageing and of old age has become an important dimension for understanding health and illness in contemporary societies (Jones and Higgs, 2010). Once confined to the edges of health care and catered for by specialties such as geriatric medicine, the challenges and problems of individual longevity have forced mainstream biomedicine to have more contact with older patients and their problems. This has meant that services and procedures have had to adapt to this situation, but in doing so, implicit understandings of what was ‘natural’ about ageing have become challenged by what has become ‘normal’ about it (Pickard, 2011). Nowhere is this more clear than in the treatment of conditions such as chronic kidney disease (CKD), where there are now many older patients and questions about what kind of services are appropriate for them have become more salient. In this article, we argue that it is important to understand how the different discourses of natural and normal ageing influence the experiences and expectations of the ‘close persons’ providing care for a person with CKD with both physical and emotional support. We assert that such discourses not only situate their satisfaction with services provided for older people with CKD but also provide an additional perspective on these engagements, one not always evident when looking at patient and providers alone.

The discourses of ageing have been described as ‘contested terrains’ by Jones and Higgs (2010) who have argued that longstanding ideas of ageing and old age as a ‘natural’ process leading to death have been radically destabilised by what is now accounted for as ‘normal’ ageing. Not only is there greater longevity, but for many individuals, there is also better health at older ages (Costa, 2005). Citing the biogerontologist Tom Kirkwood, Jones and Higgs (2010) point out that not only has there been an extension of disability-free life but that many diseases and conditions that once had a poor prognosis for older people can now be the site of successful interventions (Kirkwood, 1999). Alongside this changing notion of what is normal in later life has come the imperative to continually push the boundaries of what is possible or expected from people at different ages. This can be in terms of lifestyle, exercise, or treatment. Consequently for Jones and Higgs (2010), these different discourses are indeed ‘contested’ with some writers such as Vincent (2009), arguing for a need to return to a normatively accepted idea of natural ageing, while others see this as both an impossible and retrograde step (Mykytyn, 2009). Nicholson et al. (2012) argue that the poor health of some very old people places them in a ‘liminal’ position, ‘betwixt and between’ active living and clinically recognised dying. It is within this context that our research into the experiences of close persons caring for people with CKD on conservative management is located.

CKD is establishing itself as a global health challenge in the light of global population ageing. The range of options now available to patients with CKD of all ages has greatly increased. From a biomedical perspective, those affected by CKD will experience a gradual deterioration in kidney function and may develop chronic kidney disease stage 5 (CKD5), clinically defined as a glomerular filtration rate (GFR) <15 mL/min/1.73 m^2^ (Levey et al., 2007; The National End of Life Care Programme, 2008), where they will need renal replacement therapy (RRT) options such as dialysis and kidney transplantation if they are not to die. However, even at this stage, death is not inevitable as decline in renal function may be very slow and survival can be for many months to years. Within the field of nephrology (renal care), advances in biotechnology have seen improvements in the way that dialysis is delivered, and these have changed assumptions about age and status of older patients. When the first kidney dialysis outpatient unit was opened in 1963 in Seattle, USA, dialysis was rationed to people of working age (Blagg, 2007). Current technological advances now make it possible for dialysis to be made available to those who would have previously been seen as too old for the treatment. However, as dialysis is the only RRT option available to the majority of older patients with CKD5, given the extent of their comorbidities, it would now seem as if this option has become normalised.

At the same time, however, dialysis is a physically demanding treatment that might take a toll on some physically frail older people and may not necessarily lead to any improvement in survival or health-related quality of life (Carson et al., 2009; Murtagh et al., 2007; Smith et al., 2003). For this group of patients, the recent development of conservative kidney management (CKM) offers an alternative to dialysis. With its emphasis on both the management of symptoms associated with deteriorating kidney function and a concern for the psychosocial issues that emerge as health deteriorates (Murtagh and Sheerin, 2010), CKM is now established in several industrialised countries (De Biase et al., 2008; Morton et al., 2012; Smith et al., 2003). In the United Kingdom and Australia, it is estimated that 14% of those with CKD5 receive CKM (Carson et al., 2009; Morton et al., 2010; Murtagh, 2009). One argument raised in this article is that while CKM may be offered as an alternative to dialysis, it seems to overlap with the discourse of natural ageing insofar as treatment is replaced by management rather than being organised around active intervention. In choosing not to undertake an active treatment of dialysis, there is an implicit idea that the patients with CKD5 are accepting that they are on a relatively unpredictable trajectory towards death that is in many ways a part of what previously was seen as natural ageing. That this is an important distinction operating at both clinical and social levels is marked by the fact that in the study we are about to report the differences between management, and treatments are not always clear to those closest to the patient, while the choices are very distinct to the clinical teams who are implementing them.

In this article, we report on a multi-centre qualitative study with close persons of those with CKD5 receiving CKM in five UK renal tertiary referral centres with well-established CKM provision. By close persons we mean the individual, either a family member or friend, identified by the patient as the major provider of their physical and emotional care needs, and who is neither a volunteer nor in the employment of statutory services (Low et al., 2008). We report close persons’ past and current experience of caring for people opting for CKM, their thoughts about end-of-life care and whether current support systems adequately respond to close persons’ physical, emotional, and social needs. Unlike previous studies looking at this area (Ashby et al., 2005; Low et al., 2008; Morton et al., 2010), we will seek to show how the discourses around ageing and old age play an implicit role in shaping the experiences of this group of close persons caring for someone with CKD5 on conservative management.

## Methodology

In recruiting our sample of close persons, we consecutively approached people with CKD5 who were being conservatively managed and attending clinic in five tertiary renal centres in Southeast England between December 2009 and July 2010, to identify the key person providing the majority of their physical and emotional support. We then purposively sampled our participants for gender, age, ethnic background, their relationship to the person with CKD5, the clinical condition of the person, and the renal unit where they were being conservatively managed. Provided the person had given his or her initial consent for us to contact his or her close person, our research team approached these close persons about participation into the study. Close persons were excluded if they were caring for someone actively being considered for transplantation, were under 18 years, or acting through a voluntary agency or statutory services.

We identified 38 eligible close persons, of whom 26 gave consent to be interviewed (Table 1). Our sample were mainly women (15/26), White British (17/26) and a mean age of 63 years (SD = 14). In reflecting the age of our sample, most participants were either employed or seeking employment (18/26), but only 6 were in full-time employment. Most participants were either adult children or children-in-law (14/26), caring for people with high comorbidity requiring considerable care and with a median GFR of 10 mL/min who had been on CKM for a median of 14 months. Of the 12 who did not participate, four were too busy, two were too distressed, and three gave no reasons. A further three were not approached as the person they were caring for did not give consent.

**Table 1.** Sample characteristics of the close persons and the key demographics of person they provide care to.

Pseudonyms	Relationship with person	Age (years)	Gender	Ethnicity	Person gender	Person age (years)	Time on CKM (months)	Person eGFR
Mary	Daughter	61	Female	White British	Male	91	25	14
Ken	Husband	87	Male	White British	Female	87	5	10
Chris	Husband	71	Male	White British	Female	72	1	15
David	Husband	78	Male	White British	Female	81	17	8
Victor	Husband	81	Male	White British	Female	87	21	6
Gina	Daughter	56	Female	British Italian	Male	87	5	7
Phillip	Son	59	Male	White British	Male	87	5	8
Jan	Daughter	53	Female	White British	Female	85	27	12
Shona	Niece	42	Female	Black Caribbean	Male	91	12	12
Pam	Daughter	54	Female	White British	Female	92	31	11
George	Son	60	Male	White British	Female	96	5	8
Susan	Sister	72	Female	White British	Male	80	16	10
Rosie	Daughter	35	Female	Black Caribbean	Female	63	10	6
Nelson	Friend	51	Male	Black African	Male	61	58	14
Nick	Son	64	Male	White British	Male	89	61	10
Donna	Daughter	58	Female	Black Caribbean	Female	88	2	10
Winston	Husband	87	Male	Black British	Female	78	4	13
Doris	Wife	59	Female	White British	Male	61	20	9
Jeff	Son-in-law	64	Male	White British	Male	91	7	10
Alice	Daughter	70	Female	Black Caribbean	Female	96	16	14
Denise	Daughter-in-law	58	Female	White British	Female	80	11	10
Munisa	Daughter-in-law	38	Female	South Asian	Female	78	10	13
James	Son	64	Male	White British	Female	95	84	10
Nita	Wife	69	Female	South Asian	Male	90	23	11
Ivy	Wife	91	Female	White British	Male	93	30	12
Sandra	Friend	53	Female	White British	Female	78	14	10

The research associate (J.M.) interviewed consenting participants in a place of their choice, usually at home, and five of the interviews took place in the presence of the person with CKD5. In collecting the data, we used a narrative approach to explore: in-depth participants’ experience of caring for someone on CKM from the time of CKD diagnosis, the person’s decision about CKM, their relationship with health and social services, and how they viewed the future. We audiotaped interviews, which lasted between 20 and 90 minutes, with the participants’ consent.

All interviews were transcribed verbatim and imported into NVivo (version 8.0), a software package used to aid storage, coding, and searching of data. Transcripts were initially analysed thematically by J.M. following a chronological pattern from the diagnosis, to the everyday experience of caring, through to their future thoughts and feelings about the experience of caring for someone on CKM. We then explored the themes using a framework of ageing and disability, to see how these two concepts impacted on the experiences of close persons in caring for an elderly person with CKD5. To ensure validity and reliability, theme generation was also carried out independently by J.L. and J.M., who subsequently discussed their analysis with G.S. The main findings were brought to multi-disciplinary steering group meetings (including clinicians from nephrology and palliative care) to resolve discrepancies and reach a consensus on interpretation of texts. In presenting the results, we refer to close persons as ‘participants’ and the person with CKD5 whom the close person was providing care to as ‘the person.’

In conducting this study, we first obtained ethical approval from the Brompton, Harefield & NHLI Research Ethics Committee (09/H0708/32).

## Results

We draw on our data to explore how participants caring for a person on conservative management interacted with the themes of treatment, ageing, and death. For the purpose of analysis, we have organised the data into four specific domains as they related to the accounts of the respondents. These were (a) awareness of the onset of CKD, (b) CKM, (c) discourses of ageing in relation to health and social care, and (d) negotiating the discourse of ageing and death.

### Awareness of the onset of CKD

Most participants became informal carers of a person with CKD5 when either their relative or friend began to lose functional independence as a result of age-related frailty. Many participants had been engaged in various aspects of caring such as supporting the person with their activities of daily living or managing their complex medication before the diagnosis of CKD. Significantly, people with CKD had multiple comorbidities that accompanied the renal condition, and most participants were already receiving some support from a range of health- and social care professionals as part of their caring role.

The transition to being identified as reaching CKD5 has little specific symptomatology, but rather marks a watershed in relation to the treatment of the disease. Consequently, most participants did not perceive a dramatic change in the condition of a person with CKD5, but became aware of the fact that CKD5 was a disease of slow deterioration, which many saw as part of ageing:

She is, even with this 8 to 10% renal function, is just kind of jogging along. She’s very small, very frail, doesn’t eat a lot and somehow her body has adjusted with the aid of the medication, it adapted somehow. She doesn’t feel ill with it, she, her problem is simply confidence in walking. (James, son, 64 years)

Insofar as many of the participants felt that the condition was age related, there was also a feeling that it was their moral responsibility to provide good quality care to enable the person with CKD to remain in the person’s own home:

We have to keep everything clean just in case the health people come and say that she is not fit to live here or something, that is a worry for me. But I mean I don’t see it as that big of a problem because I see it as my job, it is my duty. She is my mother. I am African and there people stay in the house, you never put your parents in a home so I don’t want my Mother to go into a home. (Alice, daughter, 70 years)

Younger and fitter participants related to the person with CKD were able to be more engaged in providing the person with a more comprehensive level of emotional and physical support. In addition, they often acted as an intermediary between the person with CKD and professional and statutory services:

I just felt that [my father-in-law] wasn’t totally listening to what [the health professional] was saying. Anyway, like I say, they explained it [the renal condition] all to me. (Jeff, son-in-law, 64 years)

However, older participants in poor physical health had to rely on wider family networks and local neighbours to provide additional input in supporting the person with their activities of daily living, shopping, and in accessing health care.

For all participants, pressures related to how they performed their tasks and what resources could help were recurring themes. They often articulated a fear that the situation would get worse, requiring the person leave their home and to move to more sheltered accommodation. Susan, when discussing the challenges of dealing with the consequences of the persons’ deteriorating health, stated,

The first sort of thing we would go through is for him to have more help at home and see how he copes with that and should he have to go somewhere, that can’t be avoided. Obviously, he would prefer to be at home and it is something that you can’t know what is going happen, so you have got to wait until it does happen to see how you are going to cope. (Susan, sister, 72 years)

All of our participants saw the health-care problems associated with CKD as being indistinguishable from the ageing process of deterioration and decline. Consequently, their accounts are very similar to those of other carers of older people (Montgomery et al., 2007). One notable aspect of their accounts is that there is nothing specific about CKD in their experiences of caring either before or after the onset of CKD5. The only point of concern is what happens when the person with the condition needs more care than can be provided at home. There is an assumption that there will be an institutional solution. As we go on to show, CKM does not address this issue or provide a clinical pathway. Instead, the emphasis is on managing symptoms in the context of ordinary care, which assumes that death follows a natural ageing trajectory.

### Conservative kidney management

Given that most of the caring undertaken by our participants was very similar to that provided by other carers of older people, we need to address how CKM related to the care of people with CKD5. The first point that needs to be brought out is that CKM is offered as an alternative to dialysis, so that all those on it have elected to take this route. This decision is not without its problems, as it is a decision to let the disease progress in a managed way. Consequently, participants may find such a decision difficult. Not all participants were involved in the person’s decision not to choose dialysis. Those participants who felt that dialysis was not a good option did so because of the age of the person they were looking after as well as the perceived lack of benefit that dialysis would accomplish, and the disruption to their day-to-day life in contrast to the stability of the renal condition. Often, the participant would rationalise the decision from the perspective of the person, illustrated by Jan, a daughter, who had first discussed the issue of dialysis with a health professional 5 years earlier:

She (Jan’s mother) didn’t feel that it would be much quality of life (being on dialysis). I think she would just take the view that that would be the end of her life. Not that she was defeatist and gave up, but she couldn’t face the thought of being on the dialysis machine. (Jan, daughter, 53 years)

Participants’ acceptance of the person’s decision was influenced by the perception that CKM was both non-invasive and a continuation of the type of care that the person had first received following their referral to the renal teams via the low-clearance clinics. However, many participants had difficulty in defining what CKM was and had to refer back to attending outpatient renal clinics with the person to understand what CKM was trying to achieve. Moreover, CKM was seen by participants as both a more convenient and an appropriate way of managing CKD5, particularly as they did not see it affecting the person’s overall health. Acceptance of the person’s choice not to dialyse was based on the seeming inappropriateness of these biotechnologies and potential difficulties of the person preparing himself or herself for dialysis, as opposed to a specific understanding of what CKM was as one participant described:

Then they talked about dialysis and the options which would basically have been [names of two local renal units], both of which are quite inaccessible to [the person], unless she’s got me with her. Her whole feeling was, if I’ve only got this much time left, I’m not going to spend three days of every week hooked up to a machine. Home dialysis was out of the question, because her flat is absolutely tiny, so she chose the other route. (Sandra, friend, 53 years)

Some participants sought to gain greater understanding of CKM. Phillip had extensive discussions with his father about the decision whether to dialyse using Internet research:

Research has shown that people his age who have had dialysis with kidney function of around 15% live on average 3 years and maybe these people who do this conservative management may be 2 to 3 years, so there is not a huge improvement. I think that results to (the nephrologist) statement it doesn’t improve the quantity or quality. Dad is quite understanding now, that this sort of conservative management is the best. (Phillip, son, 59 years)

A few participants disagreed with the decision of the person they were caring for not to dialyse. This resulted sometimes in a tension in accepting the person’s choice of treatment. For these participants, dialysis was viewed as a life saver that could prolong the person’s life, and they felt anxious that choosing CKM would mean losing the person at an early stage of the disease progression. However, they often reluctantly accepted the choice of CKM because the option of dialysis was still available:

Well, that’s her decision I mean I have been trying to persuade her to have dialysis but it’s not my call I mean it’s up to her. It’s her life and her body, but I don’t particularly want to lose her, you know. I want her to stay with me but I mean obviously at the end of this, she is going to die if she stays as she is but they told her that the option to have dialysis at the end of this if she gets worse is still open. (Chris, husband, 71 years)

The concept of natural ageing is central to understanding participants’ acceptance of the person’s decision to be conservatively managed. For most participants, their acceptance of the decision was based on their perceptions of dialysis as an inappropriate biotechnology, disrupting the person’s lifestyle without offering any life-extending benefit. Therefore, the person’s rejection of dialysis meant that CKM was the only alternative available, which most participants accepted on the basis that CKM would cause less disruption to themselves and the person. Hence, it would initially appear that participants’ acceptance of CKM would support the notion of natural ageing, which participants acceptance of the person’s inevitable decline in health. However, the ability to reverse the decision for CKM also demonstrates the blurring of the boundaries between natural and normal ageing. For some participants, CKM is only accepted in the context of perceived stability in renal function and can be reversed if renal function deteriorates and the person wants to be dialysed.

### Discourses of ageing in relation to health and social care

For many people, the onset of CKD was initially managed by their general practitioner, up to the point that the renal functioning deteriorates to a certain level (usually when the person reaches CKD stage 4). At that point, the person was then seen by renal health professionals in ‘low-clearance’ clinics, which facilitated new relationships between participants and these professionals. The onset of CKD5 may initially have had a low impact on their experience of caring, but the person’s decision to be conservatively managed enabled a continuation of these relationships with renal health professionals who were engaging to various degrees with CKM, as a response to the complex medical regimens required to manage CKD5 conservatively.

In these relationships with renal health professionals, continuity of care was an important theme for participants. For them, it was important that they saw the same set of professionals over time, or at least to know that medical information was being shared across the renal teams and with other medical specialities. However, this often challenged the person’s multiple comorbidity. Again, while the theme of having a good relationship with professionals is one that most participants expressed, they often did not understand what CKM involved and how it would affect the person who they cared for. There was a perception that the person was receiving health care from clinicians who understood the disease trajectory and could effectively manage it. However, participants acknowledged that as non-clinicians, they were not technically skilled in differentiating between good or bad care:

Of course I wouldn’t know competence from incompetence but you understand what I mean they are extremely competent in the way that they deliver everything; very considered, soft, detailed - they are very good. I couldn’t say otherwise. (Nick, son, 64 years)

For participants, involvement with the renal team increased if the person’s renal symptoms started to deteriorate. In these cases, as part of their responsibility of managing patients conservatively, renal teams began to play a far greater role in advising both participants and the person in how to manage these symptoms. In addition, they also began to make initial arrangements for palliative care:

When final failure really comes, the renal consultant said, it would be about a month that you would have of life. We don’t know when it’ll come; we hope it will be a long way away, but when it does that’s what you need to expect. So that was good actually to kind of broach these things. The renal nurse has broached… has organized the palliative care. (James, son, 64 years)

This apparent satisfaction with how participants feel that CKM is ‘managing’ the person with renal disease is challenged by the onset of an emergency medical crisis for the person. In these situations that mainly happened outside of the hospital environment, participants spoke of their vulnerability in dealing with emergency medical situations (especially out of hours and weekends) if the person they were looking after was ill. These emergencies often challenged the notion that renal disease and medical care could be ‘managed’ with minimal medical intervention:

There has been many times when I have thought ‘what do I do now?’ Sometimes his haemoglobin levels drop ridiculously low and it happens overnight so quickly. I don’t know whether to call an ambulance or whether to take him through casualty. All these sorts of things are difficult decisions to make, as I’m not a professional. (Mary, daughter, 61 years)

The notion that the health of the person was generally stable but could deteriorate quickly meant that there was considerable uncertainty about what constituted an urgent problem. They also often felt they were not entitled to any health-care support, even with assurances from the renal team:

I know how much workload they have got and I don’t like to worry them with anything unless it is really serious for me. I could ring them up at any time and ask them a question but I just I would only do that if it was really urgent. (Mary daughter, 61 years)

Many of the difficulties faced by our participants were common to the circumstances faced by close persons caring for frail older people (Montgomery et al., 2007). For some participants, there was confusion about the management of the persons’ wider health needs which were in addition to CKD and which did not get included in CKM. Consequently, they often received conflicting medical information and un-coordinated care, as each medical speciality appeared to be treating their disease-specific symptoms without consulting each other:

He went to see the renal doctor who prescribed him some medication for blood pressure and then the cardiology doctor a week later stopped it and said he didn’t think it was good. But prior to him [the person] seeing the renal doctor when he was discharged from the hospital, cardiology actually put him on this medication. So they put him on, then they take him off then putting him on and at the moment, we are thinking who is going to give him what for his blood pressure. (Gina, daughter, 56 years)

Problems with the role and remit of social services departments were often seen as problematic. While one participant appreciated the financial advice given by social services, most struggled in getting social services to respond to the additional health needs of someone on CKM:

I was concerned when she came out [of hospital] that she wouldn’t be able to bath herself. So I phoned up the social services and they said, ‘well, we can’t tell you [about progress] until the people have seen her in hospital.’ And I said, ‘well, can you assure me that if she comes out somebody will be able to help me?’ They said, ‘oh, I can’t tell you.’ (George, son, 60 years)

CKM was developed as an acknowledgement of the need to provide appropriate care to a group of mainly elderly people with declining health, which includes support for their close persons. Our findings demonstrate that for participants, issues of continuity of care were important in much the same way that they are for many older people with health-care needs. The process of CKM although identified as a clinical intervention by professionals often failed to be perceived as that by participants who viewed it as being connected with the health-care problems of ageing.

### Negotiating the discourses of ageing and death

Nearly all participants accepted that the person’s kidneys would eventually stop functioning and could be one of the potential causes of their death. However, for most participants, the perceived stabilisation of the persons’ CKD5 on CKM meant that many were happy to continue the process of being monitored without the need to explore more about the implications of the persons’ kidney disease. For other participants, there was a lack of understanding concerning the prognosis of someone on CKM, a limited knowledge of the treatments available for CKD5 and an unwillingness for them and the person they were caring for, to discuss the future as it often led to confusion and anxiety trying to predict when the persons’ kidney function would decline and death would result.

Participants managed this uncertainty by adopting a ‘mindful’ approach focused on living in the present, while viewing the future as based on current levels of renal functioning. Indeed, a lack of medical evidence about potential future decline was used by several participants to embrace the future with feelings of hope rather than despair:

We are even planning going away next Christmas and the way I look at it, we are not worrying about it because we don’t know what is going to happen. (Ken, husband, 87 years)

Nelson did not accept the inevitability of kidney deterioration, but instead, using his own knowledge gleaned from family experience of renal disease (information not shared by those from the renal team), suggested a more positive prognosis. For this person eating a good diet could improve kidney function. As a result he saw his role as encouraging the person to eat a balanced diet more regularly and by doing so supporting the patient psychologically:

His condition can improve. No situation is stable really nothing stays the same everything hopefully changes hopefully for the better. I think it is number one priority that he needs to eat a balanced diet. Not once in a while. Not once in a blue moon. Every day. (Nelson, friend, 51 years)

Other participants felt that detailed discussions with the person they cared for about the prognosis of renal failure would highlight issues of impending death and would upset the person further:

I wouldn’t like, personally myself, I think it’d probably upset her. But she knows she’s got kidney problems that could affect her yeah. But I don’t know if it’s a good idea to tell her. (Munisa, daughter-in-law, 38 years)

However, acceptance by both participant and the person on CKM about the frailty of the latter’s health was often the key to getting both parties to discuss the future and the involvement of palliative care teams. Unfortunately, this acceptance was not sufficient to tackle issues on preference for future care if both the participant and the person had differing views on where they wanted the latter’s end-of-life care to be. For example, some participants were aware that the person they were caring for wanted to die at home, but were reluctant to support them with their wish. For these participants, past negative experiences of supporting another person die at home, either on account of the stress and anxiety it had caused, or its association with negative memories left by the dying process on the home environment made them wish that the person on CKM died in hospital or in a hospice:

Well, I think he’d like to be at home ‘cause his wife died at home, but there were two of us there (supporting the wife to die at home), he was there, and obviously he was reasonably active then, and myself. But there’s nobody there (at the person’s home), I don’t really want to do that again. I was up there three, four nights a week, and plus the days, so, I mean, I didn’t have a life for three months. (Jeff, son-in-law, 64 years)

One participant had discussed the place of death with the person they were caring for. However, this discussion seemed to favour the needs and feelings of the participant over that of the person being cared for in that it was important for the person being cared for to die in a hospice. This is because she felt that if the person died at home, it would cause too much distress and anxiety not only for herself but also her family, and that her mother’s death – the person on CKM – would be better managed in a hospice rather than at home with minimal medical intervention and expertise:

The hospice is our option [during the person’s demise] as they have the expertise and I think that it calms the anxiety and I am around people who know what they are doing. I also have a [young] son so my Mum dying here is not an option. He lives in the house and I want to keep as much trauma away from him as possible. I don’t want doctors rushing in and making him[the son] feel more anxious. (Rosie, daughter, 35 years)

For some participants, there was acceptance by both these participants and the person they were caring for about the latter’s deteriorating health. In these cases, such discussions with health-care teams had taken place about future palliative care arrangements. Nevertheless, discussions about end-of-life issues most often focused on practical issues such as funeral arrangements, rather than emotional ones:

She’s determined I should arrange her funeral before [her death]. She keeps asking me, and when she says have you done it, I know what she’s talking about. I think she has more or less accepted that she’s had a long life. (Alice, daughter, 70 years)

Our findings showed that most participants acknowledged the deteriorating nature of the persons’ health condition, but also illustrate the different ways that they negotiated this. For many, the lack of evidence for medical decline enabled them to negotiate their future to one based on hope. Some have accepted the ‘inevitable decline’ by accepting input from specialist palliative care teams and by focusing on preparing for practical tasks such as funeral arrangements. However, challenges to future preferences for care and death arise where a difference exists between the participant and the person. Our findings demonstrate that participants saw the poor health of the person as somewhere between active living and clinically recognised dying, thus supporting the concept of the ‘liminal’ position put forward by Nicholson et al. (2012).

Although one of the stated aims of CKM is the preparation of people with CKD5 for good end-of-life care, our findings suggest that discussions about preferences on future care need to be tailored depending on the readiness of participants to discuss these issues with health professionals and/or the person.

## Discussion

Our study aimed to explore the impact of age and disability on the experience of close persons caring for a person with CKD5 on CKM. Our findings would appear to support the idea that CKM is organised around the discourses of ‘natural ageing’ where later life is seen to be defined by decline and eventual death – even though this is itself becoming confused and subject to partial medicalisation (Jones and Higgs, 2010). This theme is evident in the fact that the reality of CKM for many participants is similar to the caring of other frail older people (Montgomery et al., 2007). Tensions with this discourse of natural ageing can arise because of the nature of CKD.

The choice between adopting an intervention of dialysis or accepting a strategy of CKM is the first line of tension between what has become seen as ‘normal ageing’ with its emphasis on treatment and a more passive ‘natural ageing’. Crucially, the implied nature of old age and ageing appears to intervene as a variable in how these options are thought about as well as how they influence experiences. As we have written in the earlier sections of the article, conditions such as CKD could have been seen in the past as one of the conditions associated with old age that inevitably led to death. In such circumstances, CKM would not seem to be so much a specific clinical option but rather the natural response to a biological finitude. The advances in renal medicine have changed this assumption, at least for those who are seen to be capable of benefiting from dialysis. This transformation from those with the condition being seen in terms of natural ageing to being seen as part of the creation of a new and changing normal ageing is highly problematic for those dealing with its boundaries. As the evidence of participants in this study shows the condition of the person dealing with CKD does not seem to change dramatically if not treated with dialysis. At the same time, there is a clinical awareness of the fact that it is now a terminal prognosis rather than one with a possibility of survival. It is therefore not surprising that some participants in the study are unhappy with the decisions made by the person they are providing care to. The comorbidities can be seen as part of their older lives and as things to be dealt with rather than indicators of a closing down of options. Equally, they can be seen as the reason not to add the stresses and inconveniences of dialysis to the lives of already frail individuals.

The theme of the boundary between the idea of natural ageing and the expectations of normal ageing reoccur throughout the comments of the participants. While the majority feel that they have good relations with the staff who are dealing with the CKD, it was apparent that as conditions associated with the trajectory of decline became apparent, there were difficulties with other health professionals who tended to treat the patient in relation to their various comorbid conditions that was seen to relate to their older age rather than their CKD. This is specifically so in the case of social care, where many participants’ dissatisfaction arises from the lack of procedures within social care agencies to deal responsively and flexibly to health-care problems. It is at this point that, perhaps, the real underlying nature of CKM becomes apparent.

The issue of the two discourses of ageing also emerges most poignantly in relation to our theme of planning for the future, where perhaps the combination of the uncertainty of the nature of CKM and the difficulty of dealing with the emotional issues surrounding terminal illness and death combine to create a situation where to all intents and purposes the idea of normal ageing is maintained. In line with the concepts of natural ageing, CKM is seen as presenting both resources and potential opportunities for both participants and the person to negotiate end-of-life care planning with relevant health professionals. However, unless both the participants and the person accept the terminal nature of CKD5 and have a common view about the place of end-of-life care, tensions will exist. One of the main contradictions highlighted in these findings exists between the philosophy of CKM, as supporting participants with end-of-life issues and the realities illustrated by most participants, who do not see CKM as dealing with end-of-life issues, but as a way of living for the present without the inconvenience of dialysis. Potential issues may arise as the number of symptoms increase with worsening renal functioning.

Although our article illustrates the relative satisfaction that both family members and friends had of the care provided by renal services, it also illustrated that continuity of care was needed not just in the patient journey in the management of one of their comorbid conditions, but in the management of their whole disease spectrum. More generally, however, what our study throws light on is the impact of the changing nature of later life on how health and medicine are experienced and understood. The improvements in the health of older people mean that much more can be expected of older people in terms of how they pursue the ‘will to health’ (Higgs et al., 2009) in their own lives. However, as we have also shown in this article, this also puts into stark relief those for whom the options look much more limited. For this latter group, a return to the normative structures of natural ageing has become problematic as these structures have also been destabilised by the social changes that have been brought about by a healthier older population. As Vincent (2009) points out, the loss of the normative structures that in the past helped place natural ageing in a cultural context has made it harder for people in contemporary society to negotiate illness and death as anything other than a series of health- and social care interventions. In the context of our study, it leads to the conclusion that the issues surrounding kidney disease in older patients need to be understood in the context of wider discourses of ageing and old age. By developing CKM programmes, health service planners have increasingly recognised the limitations of biomedicine in delivering acceptable health care for an increasing older and frail population. However, a major limitation of these services for both the person with CKD5 and their close person is that these services operate by focusing on a specific disease and operate on the context that ageing is not important, whereas this article shows that ageing is critically important.

Specific implications for clinical practice include two important issues, which impact on delivery of health care to a population with multiple comorbidities. First, the lack of awareness regarding the complexity of medical care delivered specifically for the renal condition was surprising. The close persons and by inference the person himself or herself did not view their CKD care as distinct from his or her other medical problems. Little comment was made about the benefit of having expert renal care although there was appreciation of the work of the renal teams. These findings illustrate the difficulties in providing seamless patient-centred care, while maintaining disease-specific treatment across multiple specialist services. The second challenging service issue identified relates to anxiety about what actions the close person take should the person get into difficulty outside of routine hours. Dedicated 24-hour helplines may help, but have not been evaluated and are likely to be costly. In addition, optimal treatment of acute events generally necessitates prompt action and temporary omission of many of the treatments designed to maintain stability under ordinary circumstances. Success depends upon timely expert knowledge, experience, clinical skill, and regular short-interval review and revision of care plans. Clearly, these key components are difficult to provide out of hours for every person with CKD5, yet they are important concerns for their close persons.

In conclusion, this article has sought to draw out the importance of ageing, both natural and now normal, for people with CKD5, and their close persons. It has shown that what might be very obvious for clinicians is not necessarily so clear for patients and others. Drawing out the implications of CKM is a challenge for clinicians if it is to avoid some of the pitfalls identified by this study.

## References

[bibr1-1363459314524805] Ashby M Hoog C Kellehear A (2005) Renal dialysis abatement: Lessons from a social study. Palliative Medicine 19: 389–396. 16111062 10.1191/0269216305pm1043oa

[bibr2-1363459314524805] Blagg AJ (2007) The early history of dialysis for chronic renal failure in the United States: A view from Seattle. American Journal of Kidney Diseases 49: 482–496. 17336711 10.1053/j.ajkd.2007.01.017

[bibr3-1363459314524805] Carson RC Juszczak M Davenport A (2009) Is maximum conservative management an equivalent treatment option to dialysis for elderly patients with significant comorbid disease? Clinical Journal of the American Society for Nephrology 4: 1611–1619. 10.2215/CJN.00510109 PMC2758251 19808244

[bibr4-1363459314524805] Costa D (2005) Causes of improving health and longevity at older ages: A review of the explanations. Genus 61: 21–38.

[bibr5-1363459314524805] De Biase V Tobaldini O Boaretti C (2008) Prolonged conservative treatment for frail elderly patients with end-stage renal disease: The Verona experience. Nephrology Dialysis and Transplantation 23: 1313–1317. 10.1093/ndt/gfm772 18029376

[bibr6-1363459314524805] Higgs P Leontowitsch M Stevenson F (2009) ‘Not just old and sick’: The will to health in later life. Ageing and Society 29: 687–707.

[bibr7-1363459314524805] Jones IR Higgs P (2010) The natural, the normal and the normative: Contested terrains in ageing and old age. Social Science & Medicine 71: 1513–1519. 20728972 10.1016/j.socscimed.2010.07.022

[bibr8-1363459314524805] Kirkwood T (1999) Time of Our Lives: The Science of Human Ageing. London: Weidenfeld & Nicolson.

[bibr9-1363459314524805] Levey AS Atkins R Coresh J (2007) Chronic kidney disease as a global public health problem: Approaches and initiatives – A position statement from Kidney Disease Improving Global Outcomes. Kidney International 72: 247–259. 17568785 10.1038/sj.ki.5002343

[bibr10-1363459314524805] Low J Smith G Burns A, et al (2008) The impact of end stage kidney disease (ESKD) on close persons: A literature review. NDT Plus 2: 67–79. 10.1093/ndtplus/sfm046 PMC5477906 28656996

[bibr11-1363459314524805] Montgomery RJV Rowe JM Kosloski K (2007) Family caregiving and congestive heart failure. Review and analysis. In: Blackburn JA Dulmus CN (eds) Handbook of Gerontology: Evidence-Based Approaches to Theory, Practice, and Policy. Hoboken, NJ: John Wiley & Sons, pp. 426–454.

[bibr12-1363459314524805] Morton RL Tong A Howard K (2010) The views of patients and carers in treatment decision making for chronic kidney disease: Systematic review and thematic synthesis of qualitative studies. BMJ 340: c12 Available at: http://www.bmj.com/highwire/filestream/343695/field_highwire_article_pdf/0/bmj.c112 (accessed 16 December 2013). 10.1136/bmj.c112 PMC2808468 20085970

[bibr13-1363459314524805] Morton RL Turner RM Howard K (2012) Patients who plan for conservative care rather than dialysis: A national observational study in Australia. American Journal of Kidney Diseases 59: 419–427. 22014401 10.1053/j.ajkd.2011.08.024

[bibr14-1363459314524805] Murtagh FEM (2009) Understanding and improving quality of care for people with conservatively-managed stage 5 chronic kidney disease – The course of symptoms and other concerns over time. PhD Thesis, King’s College London, London.

[bibr15-1363459314524805] Murtagh FEM Sheerin N (2010) Conservative management of end-stage renal disease. In: Chambers EJ Germain M Brown E (eds) Supportive Care for the Renal Patient. Oxford: Oxford University Press, pp. 253–268.

[bibr16-1363459314524805] Murtagh FEM Marsh JE Donohoe P (2007) Dialysis or not? A comparative survival study of patients over 75 years with chronic kidney disease stage 5. Nephrology Dialysis and Transplantation 22: 1955–1962. 10.1093/ndt/gfm153 17412702

[bibr17-1363459314524805] Mykytyn CE (2009) Anti-aging is not necessarily anti-death: Bioethics and the front lines of practice. Medicine Studies 1: 209–228.

[bibr18-1363459314524805] Nicholson C Meyer J Flatley M (2012) Living on the margin: Understanding the experience of living and dying with frailty in old age. Social Science & Medicine 75: 1426–1432. 22800918 10.1016/j.socscimed.2012.06.011

[bibr19-1363459314524805] Pickard S (2011) Health, illness and normality: The case of old age. BioSocieties 6: 323–341.

[bibr20-1363459314524805] Smith C Da Silva Gane M Chandna S (2003) Choosing not to dialyse: Evaluation of planned non-dialytic management in a cohort of patients with end-stage renal failure. Nephron Clinical Practice 95: c40–c46. 14610329 10.1159/000073708

[bibr21-1363459314524805] The National End of Life Care Programme (2008) End of life care in advanced kidney disease: A framework for implementation. Available at: http://www.ncpc.org.uk/sites/default/files/EndOfLifeCareInAdvancedKidneyDisease.pdf (accessed 25 February 2014).

[bibr22-1363459314524805] Vincent J (2009) Ageing, anti-ageing, and anti-anti-ageing: Who are the progressives in the debate on human biological ageing. Medicine Studies 1: 197–208.

